# The cytochrome P450 family in the parasitic nematode *Haemonchus contortus*

**DOI:** 10.1016/j.ijpara.2014.12.001

**Published:** 2015-03

**Authors:** Roz Laing, David J. Bartley, Alison A. Morrison, Andrew Rezansoff, Axel Martinelli, Steven T. Laing, John S. Gilleard

**Affiliations:** a University of Glasgow, Glasgow, UK; b Moredun Research Institute, Edinburgh, UK; c University of Calgary, Calgary, Canada; d Welcome Trust Sanger Institute, Cambridge, UK

**Keywords:** Parasite, Nematode, Metabolism, Cytochrome P450, Gene expression, Resistance

## Abstract

*Haemonchus contortus*, a highly pathogenic and economically important parasitic nematode of sheep, is particularly adept at developing resistance to the anthelmintic drugs used in its treatment and control. The basis of anthelmintic resistance is poorly understood for many commonly used drugs with most research being focused on mechanisms involving drug targets or drug efflux. Altered or increased drug metabolism is a possible mechanism that has yet to receive much attention despite the clear role of xenobiotic metabolism in pesticide resistance in insects. The cytochrome P450s (CYPs) are a large family of drug-metabolising enzymes present in almost all living organisms, but for many years thought to be absent from parasitic nematodes. In this paper, we describe the CYP sequences encoded in the *H. contortus* genome and compare their expression in different parasite life-stages, sexes and tissues. We developed a novel real-time PCR approach based on partially assembled CYP sequences “tags” and confirmed findings in the subsequent draft genome with RNA-seq. Constitutive expression was highest in larval stages for the majority of CYPs, although higher expression was detected in the adult male or female for a small subset of genes. Many CYPs were expressed in the worm intestine. A number of *H. contortus* genes share high identity with *Caenorhabditis elegans* CYPs and the similarity in their expression profiles supports their classification as putative orthologues. Notably, *H. contortus* appears to lack the dramatic CYP subfamily expansions seen in *C. elegans* and other species, which are typical of CYPs with exogenous roles. However, a small group of *H. contortus* genes cluster with the *C. elegans* CYP34 and CYP35 subfamilies and may represent candidate xenobiotic metabolising genes in the parasite.

## Highlights

• The *Haemonchus contortus* genome encodes a large family of cytochrome P450 (CYP) genes.
• *Haemonchus contortus* lacks the dramatic CYP family expansions seen in *Caenorhabditis elegans*.
• *Haemonchus contortus* orthologues of *C. elegans* CYPs share similar expression profiles.
• The majority of *H. contortus* CYPs are most highly expressed in larval stages.
• The parasite intestine is a major site of CYP expression.

## Graphical abstract

**Figure:**
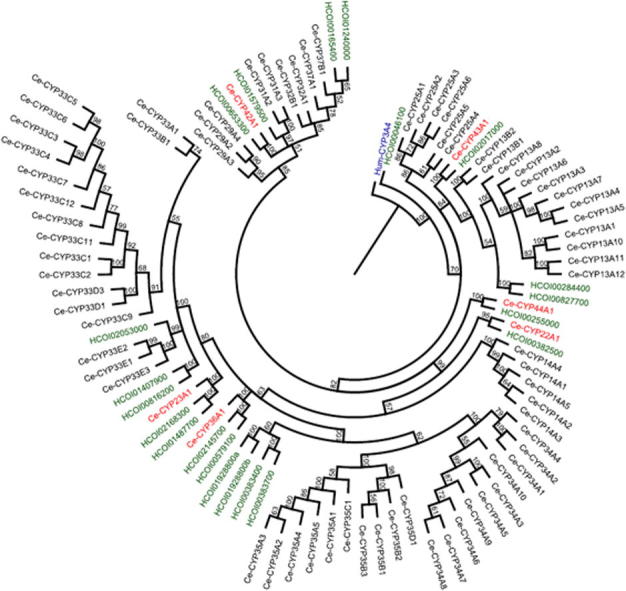

## 1 Introduction

Anthelmintic resistance is a major threat to the sheep industry worldwide and is an emerging concern for parasite control in other species (Kaplan, 2004). The mechanisms underlying resistance in parasitic nematodes are not fully understood and there is a lack of sensitive diagnostic tools or methods to study the evolution of resistance or the impact of different control strategies.

Cytochrome P450s (CYPs) are a large superfamily of enzymes found in almost all living organisms (Nelson et al., 1993); their ubiquity may reflect their ancient origin and physiological importance. CYPs catalyse a wide range of reactions involving both endogenous and exogenous substrates; they are involved in the biosynthesis and catabolism of steroids, retinoids, prostaglandins and fatty acids, and the detoxification of exogenous substrates including drugs and insecticides (Nelson et al., 1993; Mansuy, 1998; Nebert and Dieter, 2000; Anzenbacher and Anzenbacherova, 2001; Nebert and Russell, 2002; Thomas, 2007). CYPs are the main enzymes involved in phase I metabolism, which increases the solubility of a substrate, usually by adding or uncovering a hydrophilic group. This is followed by phase II metabolism, catalysed by uridine dinucleotide phosphate glucuronosyl transferases (UGTs) and GSTs, which conjugate the metabolite for excretion. The human genome encodes 57 CYPs, of which five are responsible for the metabolism of approximately 75% of drugs in clinical use (Williams et al., 2004; Wienkers and Heath, 2005) and one enzyme, CYP3A4, catalyses more than 50% of those reactions (Smith and Jones, 1992; Fujita, 2004).

In insects, the association of CYP expression with drug resistance is well established. In 2001, Daborn et al. found overexpression of a single gene, *cyp6g1*, was responsible for multi-insecticide resistance in 20 field strains of *Drosophila melanogaster*. Work by Schlenke and Begun (2004) then demonstrated that overexpression of *cyp6g1* also conferred insecticide resistance in *Drosophila simulans.* The mechanism of *cyp6g1* upregulation in both cases was a transposable element insertion in the 5′ flanking sequence; an *Accord* transposon in *D. melanogaster* and a *Doc* transposon in *D. simulans.* Mutations affecting eight additional CYPs have since been shown to confer insecticide resistance in *Drosophila* (Maitra et al., 1996; Amichot et al., 2004; Pedra et al., 2004; Bogwitz et al., 2005; Daborn et al., 2007). In field populations of *Anopheles* mosquitoes, pyrethroid resistance has been associated with overexpression of *cyp6p9* in *Anopheles funesus* (Amenya et al., 2008) and *cyp6z1*, *cyp6p3* and *cyp6m2* in *Anopheles gambiae* (Nikou et al., 2003; Djouaka et al., 2008).

For many years, it was suggested that oxidative metabolism was not a major process in parasitic nematodes and CYP activity was generally absent, or at a low level, in parasite extracts (Pemberton and Barrett, 1989; Precious and Barrett, 1989; Barrett, 1998, 2009; Yadav et al., 2010). However, there is now strong evidence that this is not the case. The genome of *Caenorhabditis elegans* encodes 80 CYPs. The functions of most are unknown, but a number are induced with exposure to xenobiotics (Menzel et al., 2001, 2005). The benzimidazole anthelmintic, albendazole (ABZ), induces the expression of *cyp35a2*, *cyp35a5* and *cyp35c1* in *C. elegans* and is metabolised to two ABZ-glucoside metabolites and a possible ABZ-sulphoxide metabolite, consistent with phase I and phase II metabolism (Laing et al., 2010). Drug metabolism in parasitic nematodes has not been widely studied, but it is likely that these biotransformation pathways are conserved. CYP oxidase activity has been demonstrated in microsomal preparations of *Haemonchus contortus* (Kotze, 1997) and the parasite produces the same ABZ metabolites as *C. elegans* (Cvilink et al., 2008). Interestingly, benzimidazole-resistant and multi-drug-resistant *H. contortus* isolates have been shown to produce greater quantities of flubendazole metabolites than a susceptible isolate, suggesting that increased activity of detoxification enzymes are associated with anthelmintic resistance. Despite being metabolised by CYP3A in humans (Zeng et al., 1998), ivermectin (IVM) metabolism has not been detected in *C. elegans* (Laing et al., 2012) or *H. contortus* (Vokral et al., 2013). However, the CYP inhibitor piperonyl butoxide (PBO) has recently been found to potentiate the effect of IVM in larval stages of *Cooperia oncophora* and *Ostertagia ostertagia*, and to reverse IVM resistance in larval stages of *C. oncophora* (AlGusbi et al., 2014). PBO has also been shown to increase the toxicity of a pesticide, rotenone, to *H. contortus* adults and larvae (Kotze et al., 2006).

The present study describes the genetic characterisation of the *H. contortus* CYP family. All CYPs are single domain proteins which facilitates their discovery using homology-based methods, but their propensity to expand by duplication can generate clusters of highly similar genes, pseudogenes and detritus exons (Tijet et al., 2001; Nelson et al., 2004; Thomas, 2006, 2007; Baldwin et al., 2009). This provides a challenge for the comprehensive annotation and global analysis of CYP family gene expression in organisms which lack a high quality finished genome sequence. We addressed this potential barrier for *H. contortus* by designing a quantitative real-time PCR (qPCR) assay for short coding sequence “tags” from every CYP fragment identified in the early *H. contortus* genome assemblies. There was an anticipated redundancy in this approach since highly fragmented genes would have multiple tags and since early genome assemblies were generated from multiple worms, and would potentially contain numerous allelic variants. However, once a good quality *H. contortus* draft genome and transcriptome were available (Laing et al., 2013), it was possible to undertake RNA-seq expression analysis. This was then validated with the real-time data from the corresponding CYP tags. In this paper we present the characterisation and global expression analysis of the *H. contortus* cytochrome P450 gene family using this dual approach.

## 2 Materials and methods

### 2.1 Identification of CYP sequence

*Haemonchus contortus* genome assemblies were produced by the Wellcome Trust Sanger Institute, UK (http://www.sanger.ac.uk/resources/downloads/helminths/haemonchus-contortus.html). Polypeptide sequences for all *C. elegans* CYPs listed in Wormbase were used to search the 93 Mb *H. contortus* assembled contigs 27/01/06 database using tBLASTn. This search was repeated with CYPs of interest from other species: human CYP3A4 and *D. melanogaster* CYP6G1 polypeptides. All contigs identified with significance of *P* < 0.005 were used for a BLASTx reciprocal search of the *C. elegans* Wormpep database and the closest matched protein for each was recorded. This procedure was repeated for the revised 214 Mb *H. contortus* assembled contigs 12/11/07 database and 279 Mb *H. contortus* assembled contigs and supercontigs 21/08/08 databases as they became available. Each *H. contortus* CYP sequence identified with a previous search was used for a search (BLASTn) of each new database for completeness and to maintain continuity with the nomenclature. The *H. contortus* homologue of *C. elegans ama-1,* which encodes a subunit of RNA polymerase II, was identified bioinformatically for use as an internal control to normalise the qPCR expression assays (*Hc-ama*; GenBank Accession number CDJ91461).

Gene models encoding CYP domains were identified in the *H. contortus* draft genome (GenBank Accession numbers CDJ81012.1, CDJ82292.1, CDJ82330.1, CDJ83273.1, CDJ83276.1, CDJ84496.1, CDJ84541.1, CDJ84895.1, CDJ86031.1, CDJ87159.1, CDJ92488.1, CDJ92489.1, CDJ92489.1, CDJ94176.1, CDJ94556.1, CDJ95149.1, CDJ96396.1, CDJ96745.1, CDJ97632.1, CDJ97895.1, CDJ97964.1, CDJ97972.1, CDJ98403.1, CDJ98491.1, HCOI01487700 and HCOI01240000) and manually curated (Supplementary Table S1). Conceptual translations of all CYP tags were also used to search (tBLASTn) the draft genome scaffold database. Primers from the real-time screen were used to search (BLASTn) the gene model and scaffold databases to correlate the results of the real-time screen with CYP genes and CYP loci in the draft genome.

For phylogenetic analyses, conceptual translations of *H. contortus* and *C. elegans* CYPs were aligned in Geneious version 6.1.2 (http://www.geneious.com) and a neighbour-joining tree was generated using a Jukes-Cantor genetic distance model, with human CYP3A4 (GenBank Accession number AAF21034) as an outgroup. Software from Interpro (http://www.ebi.ac.uk/interpro/) was used for functional analysis of predicted proteins.

### 2.2 *Haemonchus contortus* maintenance and culturing

Experimental infections were performed at the Moredun Research Institute, UK as described previously (Laing et al., 2013). All experimental procedures were examined and approved by the Moredun Research Institute Experiments and Ethics Committee (MRI reference E46/11) and were conducted under approved British Home Office licenses in accordance with the Animals (Scientific Procedures) Act of 1986. The *H. contortus* isolates used in this study are susceptible to all broad spectrum anthelmintic drugs. The MHco3(ISE) isolate (Roos et al., 2004) was used for all comparisons in the qPCR screen other than the soma (worms bodies with intestine removed) and intestine comparison, which used cDNA from the Beltsville isolate (as described in Rehman and Jasmer, 1998), kindly provided by Prof. D. Jasmer (Washington State University, USA). The inbred MHco3(ISE).N1 isolate (Sargison, N., 2009. Development of genetic crossing methods to identify genes associated with macrocyclic lactone resistance in the sheep nematode parasite, *Haemonchus contortus* (PhD thesis). University of Edinburgh, UK) was used for all RNA-seq libraries, other than the female intestine samples, which were made from the MHco3(ISE) isolate using the same dissection technique as for the Beltsville isolate (Rehman and Jasmer, 1998). The generation and analysis of all RNA-seq libraries has been described previously (Laing et al., 2013) and the remainder of the methods relate to the qPCR screen.

Three biological replicates (material from different populations of worms from different donor sheep) of each lifecycle stage were used for the L3, L4 and adult comparisons, and three technical replicates were used for the male and female comparison (different groups of worms isolated from the same donor sheep). In the case of the soma and intestine comparison, no replicates were possible due to the limited material generated from dissections.

### 2.3 Sample preparation

RNA was extracted using a standard Trizol procedure (Life Technologies, UK, 15596-026). RNA samples were quantified by 260/280 nm absorption on a Gene Quant Pro spectrophotometer (Amersham Biosciences, UK) and analysed by gel electrophoresis. First-strand cDNA was synthesised from 5 μg of total RNA using random hexamer primers, following the manufacturer’s protocol (Cloned AMV First-strand cDNA Synthesis Kit, Life Technologies, 12328-040). Residual primers, nucleotides and enzymes were removed from the cDNA with a PCR purification kit (QIAquick PCR Purification Kit, QIAGEN, UK, 28106) following the manufacturer’s instructions.

### 2.4 qPCR screen

Primers were designed with predicted melting temperatures of 60 ± 2 °C, lengths of 18–20 nucleotides (nt), and GC contents of >45%. Where possible, amplicons of 160–200 bp (range 120–212 bp) were designed to span introns. Supplementary Table S2 shows primer sequences. Primer pairs were tested with PCR amplification of L3 and adult cDNA. A single band of the expected size when run on an agarose gel was taken as a positive result. This was confirmed with melting curve analysis by the qPCR machine in each real-time experiment.

The low expression level of the majority of the CYP tags prevented the use of standard curves as a measure of efficiency, as threshold was only reached in the most concentrated samples in a serial dilution. Standard curves were run for the control gene *Hc-ama* to confirm its efficiency (*R*^2^ = 1.00, *E* = 102.7%) and linear regression on the fluorescence during the exponential phase of the PCR was used to estimate amplification efficiency (*E*) of all CYP primer pairs using the computer software LinRegPCR (Ramakers et al., 2003). Fifty-three primer pairs had *E* > 0.90, five had *E* = 0.70–0.90 and 10 had *E* < 0.70 (the latter group included six tags for which adult expression was only barely detected).

All reactions were carried out using a Stratagene Mx3000P QPCR system. Brilliant SYBR Green QPCR master mix (Stratagene, UK, 600548) was used with a final concentration of primers between 300 and 400 nM in a total reaction volume of 25 μl. Data were captured and analysed using Stratagene MxPro software. Relative expression was calculated using the ΔΔCT method (Livak and Schmittgen, 2001), using *Hc-ama* as the internal control. Significant differences between samples were identified with the Student’s *t*-test at *P* < 0.05.

## 3 Results

Seventy-three partial CYP sequences were identified on 61 supercontigs in the *H. contortus* genome assembly databases from 2006 to 2008. In most cases, the supercontigs were too short to encode full-length CYPs, with the majority covering less than 20% of the gene. Sixty-eight of the CYP sequences were amenable to primer design and amplified a product from *H. contortus* L3 or adult cDNA, and were included in the qPCR assay.

**Fig. 1:**
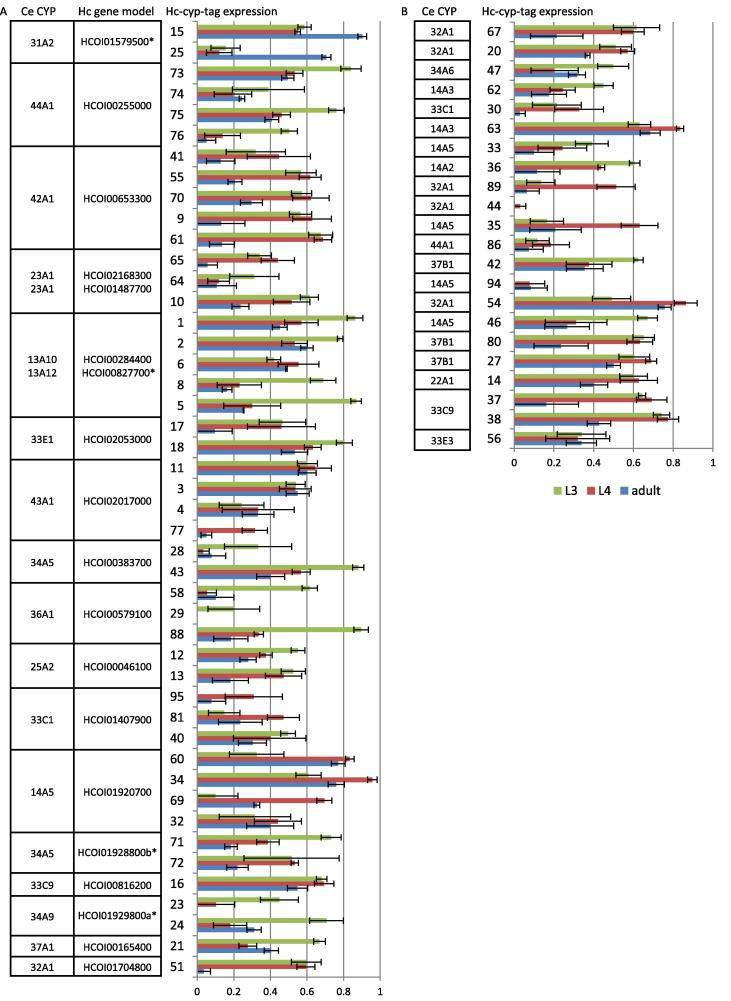
Quantitative real-time PCR (qPCR) assay of cytochrome P450 (CYP) tag expression in L3, L4 and adult *Haemonchus contortus* (Hc). Tags representing the same gene model are clustered in (A), together with the predicted *Caenorhabditis elegans* (Ce) homologue. Orphan tags that do not align to a gene model are in (B). The figure summarises expression data derived from two real-time experiments (L3 versus adult and L4 versus adult), each run in triplicate. Expression levels are normalised to *H. contortus ama-1*. Error bars show S.E.M. Primers aligned with less than 100% identity to four gene models (HCOI01637300 (Hc-cyp-tag20), HCOI00383400 (Hc-cyp-tag28 and Hc-cyp-tag43), HCOI02145700 (Hc-cyp-tag47), HCOI01240000 (Hc-cyp-tag55)) and no primers aligned to HCOI00576400 or HCOI00382500 (these gene models are not shown). Gene models with asterisks have undergone manual curation (Supplementary Table S1).

Twenty-five CYP gene loci were identified in the *H. contortus* draft genome; 16 full-length genes and nine partial sequences. RNA-seq was used to compare their expression in different life-stages, sexes and the parasite gut. These findings were validated with expression data generated with the qPCR screen for the 68 CYP tags. Forty-six CYP tags aligned to the 25 CYPs in the draft genome, of which two partial CYP sequences (HCOI00576400 and HCOI00382500) had no tags aligned. Three tags (Hc-cyp-tag1, Hc-cyp-tag2 and Hc-cyp-tag6) aligned to two different gene models (HCOI00284400 and HCOI00827700); their primers are expected to amplify both genes. Twenty-two ‘orphan’ CYP tags contained multiple non-synonymous single nucleotide polymorphisms (SNPs) when aligned to the draft genome (Fig. 1).

### 3.1 CYP expression differs in parasite lifecycle stages, sexes and tissues

**Fig. 2:**
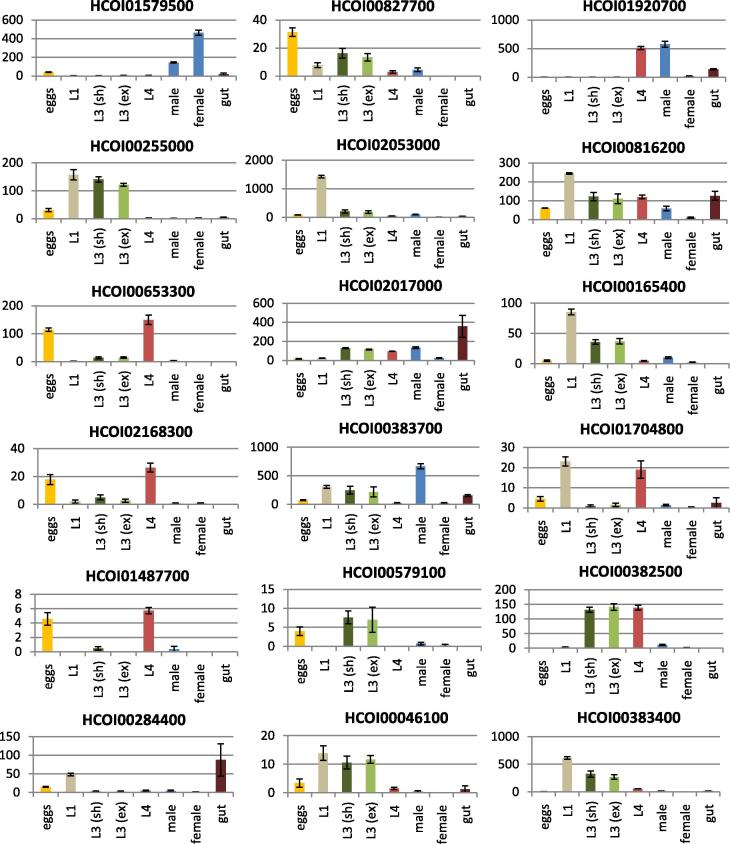
RNA-seq cytochrome P450 (CYP) expression. Read numbers are normalised to effective library size and error bars show the S.E.M. for three replicates. No reads mapped to gene models HCOI01407900, HCOI01928800a, HCOI01928800b, HCOI01637300, HCOI02145700, HCOI00576400 or HCOI01240000 and those are not shown. Data is included for both sheathed (sh) and exsheathed (ex) L3s.

Constitutive CYP expression was compared in a range of *H. contortus* life-stages with RNA-seq (Fig. 2), including eggs, L1s, sheathed L3s, exsheathed L3s, L4s and adults. Overall CYP expression was relatively low in all stages, but the expression levels of individual CYPs varied dramatically throughout the parasite lifecycle, giving many genes a distinct expression profile. The majority of CYPs had highest expression in one or more of the four larval stages examined, but a small number showed higher expression in the egg or in the adult. The RNA-seq data generally corresponded well with results from the qPCR screen (Fig. 1), but genes with very low expression in one of the stages tended to be more variable between replicates. Unsurprisingly, CYPs with a difference in male and female expression varied more between the RNA-seq data and the real-time life-stage data as the latter used pooled unsexed adults, so a separate comparison of male and female expression using the real-time screen was also undertaken (Supplementary Fig. S1A).

RNA-seq showed the majority of CYP genes were more highly expressed in the adult male than the female, except for HCOI01579500, which was more highly expressed in the female (Fig. 2). This was consistent with results of the qPCR screen where 25 CYP tags were more highly expressed in the male sample at *P* < 0.05 but only Hc-cyp-tag15 and Hc-cyp-tag25 (which correspond to gene model HCOI01579500) were more highly expressed in the female at *P* < 0.05.

RNA-seq analysis of the adult gut showed a number of CYPs were enriched in the worm intestine (Fig. 2) including HCOI02017000, HCOI00816200, HCOI00383700, HCOI00284400 and HCOI01920700. For the qPCR screen we compared the adult intestine with adult soma (worm bodies with the intestines removed) (Supplementary Fig. S1B). The results generally corresponded well with the RNA-seq findings, with the majority of CYP tags more highly expressed in the intestine. The exceptions were tags representing HCOI01579500 (Hc-cyp-tag15, Hc-cyp-tag25), HCOI00255000 (Hc-cyp-tag73, Hc-cyp-tag74, Hc-cyp-tag75, Hc-cyp-tag76) and HCOI01407900 (Hc-cyp-tag40, Hc-cyp-tag81, Hc-cyp-tag95), which had higher expression in the soma. Five CYP tags representing gut-expressed HCOI00284400 showed conflicting expression levels in the real-time screen, but some of these tags were expected to also amplify HCOI00827700, as described previously, which does not appear to be enriched in the intestine.

### 3.2 Comparative analysis with the *C. elegans* CYP family

**Fig. 3:**
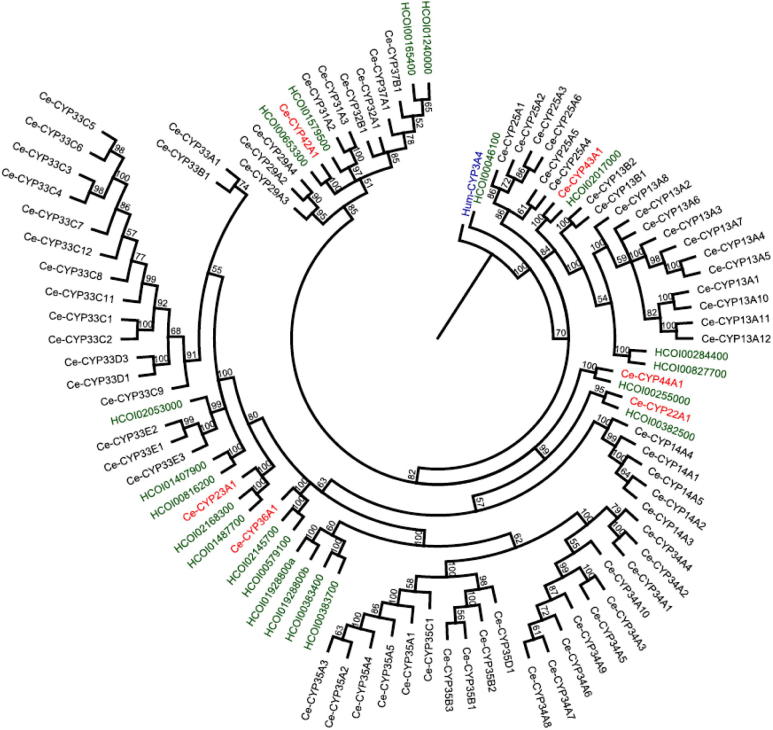
Neighbour joining tree of *Haemonchus contortus* and *Caenorhabditis elegans* cytochrome P450s (CYPs). Conceptual translations of *H. contortus* gene models (green) and *C. elegans* CYPs, rooted to human CYP3A4 (blue). Putative orthologues for all *C. elegans* single-member CYPs (red) are present in *H. contortus* but the parasite appears to lack the dramatic expansions seen in many *C. elegans* subfamilies.

Fig. 3 shows a neighbour-joining tree of conceptual translations of the *H. contortus* CYP gene models aligned with all CYP polypeptides in *C. elegans*. The *C. elegans* genome encodes 80 CYPs of which six are single-member genes. In a survey of 10 vertebrates, Thomas (2007) found that all CYP genes could be classified as either phylogenetically stable or phylogenetically unstable. He found that CYPs with known endogenous functions were more likely to be stable, with no or few gene duplications or losses, while CYPs with a role in xenobiotic metabolism were most commonly found in unstable gene clusters, proposed to have arisen through local gene duplication.

Homologues for all single-member CYPs in *C. elegans* are present in *H. contortus* (Fig. 3) and the majority are also single copy genes in the parasite. The exceptions are *C. elegans* CYP23A1 and CYP36A1, which each share homology with two gene models in *H. contortus*, but these may reflect allelic sequence in the draft genome. A number of single-member CYPs share similar expression profiles in both nematodes, supporting their classification as putative orthologues. For example, *C. elegans cyp22a1* (*daf-9*) is involved in promoting reproductive development and regulating the dauer pathway (Gerisch and Antebi, 2004). It is expressed in hypodermal and neuronal cells. Life-stage expression is highest in the larval stages, particularly L2 and L3, with low expression in the embryo and adult stages. The putative orthologue in *H. contortus*, HCOI00382500, has highest expression in the L3 and L4 stages and low expression in the egg, L1 and adult, and is not expressed in the gut.

*Caenorhabditis elegans cyp31a2* and *cyp31a3* encode two highly similar proteins (94.5% identity) involved in the formation of lipids required for eggshell production (Benenati et al., 2009). The genes are expressed in gonads, oocytes and embryos, and their proteins have been shown to function with *C. elegans* cytochrome P450 reductase, EMB-8 (see Section 3.3). The conceptual translation of one parasite gene model, HCOI01579500*,* shares 57% amino acid identity with both CYP31A2 and CYP31A3 (Supplementary Fig. S2). HCOI01579500 had a characteristic expression profile that was notably different from the majority of other *H. contortus* CYPs. It was the most highly expressed CYP identified in adult worms, showed higher expression in the female than the male, and higher expression in the body than the intestine. Interestingly, the gene assembled from sequence in earlier genome databases (from Hc-cyp-tag15 and Hc-cyp-tag25) encodes a polypeptide with two amino acid substitutions relative to HCOI01579500 in the draft genome, but it is unclear whether this represents a paralogue, as in *C. elegans*, or a different allele.

Strikingly, *H. contortus* seems to lack the dramatic gene expansions seen in many *C. elegans* CYP families, predicted to comprise the ‘unstable’ environmental response genes (Fig. 3). In some cases there appears to be a single homologous family member in the parasite, for example HCOI02053000 clusters with the three-member *C. elegans* CYP33E subfamily, but *H. contortus* doesn’t seem to have undergone the extensive gene duplications that have generated the multiple large CYP “blooms” described in other species (Feyereisen, 2010). Only two parasite CYPs cluster with the 12 member *C. elegans* CYP13A subfamily and none cluster with the 15 member CYP33 family. The only apparent expansion consists of four parasite CYPs which cluster with the 20 *C. elegans* CYPs in the CYP34 and CYP35 families, and appears to have arisen through two gene duplications: HCOI01928800b lies directly downstream of HCOI01928800a on scaffold_930 and HCOI00383700 lies two genes downstream of HCOI00383400 on scaffold_1500. In *C. elegans*, members of the CYP13A, CYP33C, CYP33E and CYP35A subfamilies are inducible with xenobiotics (Menzel et al., 2001, 2005).

### 3.3 Functional and regulatory pathways are conserved in nematodes

*Caenorhabditis elegans emb-8* is thought to encode the orthologue of NADPH-cytochrome P450 reductase, a protein that supplies electrons to cytochrome P450 enzymes. The *H. contortus* draft genome encodes a putative *emb-8* orthologue (GenBank Accession number CDJ82588.1) and the conceptual translation shares 69% amino acid identity with the *C. elegans* protein (Supplementary Fig. S3) and 89% identity with the *Necator americanus* protein. *Haemonchus contortus* EMB-8 has a conserved NADP cytochrome P450 reductase domain, containing all 40 expected residues for the associated NADP binding pocket, flavin adenine dinucleotide (FAD) binding pocket, FAD binding motif, phosphate binding motif, beta–alpha–beta structure motif and the four expected catalytic residues. RNA-seq data shows that, similar to *C. elegans emb-8*, the parasite gene is expressed in all life-stages. This is supported by PCR amplification (to obtain the full-length coding sequence (CDS) in earlier genome assemblies; data not shown) from the L3 and adult stages.

The *C. elegans* genome encodes 284 nuclear receptor genes, of which three have been predicted to reside in the same subfamily as the mammalian pregnane X receptor (PXR) and constitutive androstane receptor (CAR) proteins known to regulate the expression of xenobiotic metabolising CYPs: *daf-12*, *nhr-8* and *nhr-48* (reviewed in Xu et al., 2005). Eighty-three gene models in the *H. contortus* draft genome encode nuclear hormone receptor domains and while clear homologues are not apparent, all isoforms of *C. elegans daf-12*, *nhr-8* and *nhr-48* share most sequence similarity with two *H. contortus* gene models (GenBank Accession numbers CDJ84776.1 and CDJ92018.1).

## 4 Discussion

This work was originally based on sequences from the 2006–2008 *H. contortus* genome assemblies. These assemblies were generated from genomic DNA isolated from multiple adult worms of the anthelmintic-susceptible MHco3(ISE) isolate and the CYP family remained highly fragmented. This may partly reflect their large size and high number of intron insertions. Since genes in the same subfamilies could potentially be more similar than allelic variants of the same gene, ambiguous partial CYP sequences “tags” were left unassembled and used to develop a qPCR screen. The CYP tag sequences and their corresponding primers were aligned to the draft *H. contortus* genome, which was generated from a single male worm, to correlate results with full-length CYP genes. Twenty-two CYP tags contained multiple non-synonymous SNPs when aligned to the draft genome. There are a number of possible explanations for this: they may represent allelic variants of CYP genes; CYPs can be subject to copy number variation and these may represent copies not present in the individual sequenced for the draft genome; or some CYP loci, perhaps containing recent duplications, may remain unassembled or have been forced to a consensus sequence. Ongoing improvements to the reference genome, combined with re-sequencing of individual worms, should clarify the origin of these tags.

The results of the qPCR screen were largely consistent with RNA-seq analysis, demonstrating the value of a sequence tag approach, particularly for complex gene families in organisms without good draft or finished genomes. CYP expression was generally highest in one or more larval stages with only a small subset of CYPs showing higher adult expression. These findings are consistent with work by Kotze (1997) where mono-oxygenase activity in microsomal preparations from three *H. contortus* life stages was measured, identifying high activity in L1 and L3 larvae, with lower activity in adults. It is possible that the free-living larval stages are exposed to a wider range of environmental toxins than the adult and thus may require a higher CYP activity to detoxify exogenous compounds. Alternatively, there may be a greater requirement for the metabolism of endogenous toxins and compounds essential for development in larval stages.

It has also been suggested that the decrease in *H. contortus* CYP activity from L3 to adult could parallel a transition from aerobic metabolism outside the host to anaerobic metabolism inside (Kotze, 1997). Interestingly, the results of the current study found relatively high CYP expression in *H. contortus* L4 and adults for a small subset of genes. This would suggest the abomasal environment does not prohibit CYP activity, as does the adult expression of NADPH-cytochrome reductase. One hypothesis is that the parasite could derive an adequate supply of molecular oxygen from the host blood supply to facilitate CYP activity. An extreme example of this would be the high levels of CYP activity detected in microsomal preparations from adults of the blood fluke *Schistosoma mansoni* (Saeed et al., 2002). Adults of the hookworm *Nippostrongylus brasiliensis* reside in the small intestine, yet also maintain a functional aerobic respiratory chain, relying on oxygen as the terminal electron acceptor (Fry and Jenkins, 1983, 1984). This is facilitated by their attachment to the gut mucosa, where oxygen tensions are higher than in the lumen. *Haemonchus contortus* adults, which attach to the abomasal mucosa, were also shown to be capable of both aerobic and anaerobic respiration (Fry and Jenkins, 1984). Consistent with this, it was hypothesised that CYPs might catalyse both aerobic and anaerobic pathways of metabolism in *H. contortus*. In 1999, Kotze showed that *H. contortus* L3s were capable of peroxide-supported CYP activity independent of a supply of molecular oxygen in vitro. However, the results for adults were inconclusive; in vitro oxidase activity was demonstrated in microsomal preparations from adult worms, but was inhibited by both CYP and peroxidase inhibitors.

A number of CYPs appear to be more highly expressed in adult *H. contortus* males than females. In *D. melanogaster*, CYPs have been associated with male behavioural phenotypes, with expression of *cyp6a20* linked to male aggressive behaviour in an inducible and reversible manner (Wang et al., 2008) and with *cyp4d21* facilitating mating in adult male flies (Fujii et al., 2008).

A number of CYPs are expressed in the parasite intestine, which is consistent with the hypothesis that the intestine is the prime site of detoxification in nematodes (An and Blackwell, 2003; McGhee, 2007). In *D. melanogaster*, most CYPs are expressed in the midgut, Malpigian (renal) tubules and fat body, which constitute the main organs of detoxification in insects (Chung et al., 2009) and in *C. elegans* a number of xenobiotic inducible CYPs are expressed in the intestine including *cyp13a7*, *cyp14a3*, *cyp33c2*, *cyp33e2* and *cyp35a2* (Menzel et al., 2001; Chakrapani et al., 2008). The gut-enriched CYPs in *H. contortus* may therefore represent a subset of candidate xenobiotic metabolising genes in the parasite, but it is also possible that some xenobiotic metabolising CYPs would have low constitutive expression in the gut until induced by their substrate. Conversely, HCOI02017000 was the most highly expressed CYP in the intestine, but as a single member CYP, by definition is more likely to have an endogenous role. The *C. elegans* orthologue is also enriched in the gut (Pauli et al., 2006), but the gene function is unknown.

Preliminary work comparing constitutive and anthelmintic-induced CYP expression in MHco3(ISE) with two resistant isolates, MHco4(WRS) and MHco10(CAVR), failed to identify any statistically significant differences in CYP gene expression (data not shown). However, further work is required to control for the notable between-isolate variation, which may in part be due to low levels of CYP expression in calibrator samples and high levels of CYP gene polymorphism between strains, and to compare the effects of different drug concentrations on CYP induction.

Comparative analysis with *C. elegans* was used to identify putative CYP orthologues with similar constitutive expression patterns in *H. contortus*. The *C. elegans* single-member CYPs appear to be highly conserved, suggesting they may perform essential housekeeping roles. Notably, *H. contortus* appears to lack the dramatic CYP family expansions seen in *C. elegans*, which are predicted to occur in CYPs with environmental response roles and include those induced on exposure to xenobiotics. It is conceivable that highly identical CYPs, such as those generated by recent duplications and clustered in the genome, a characteristic of CYP blooms, could remain unassembled in the *H. contortus* genome or could be forced into a consensus assembly. However, the lack of parasite sequence with homology to some of the largest *C. elegans* subfamilies, including the orphan CYP tags (which might be considered to cast a broader net), suggests they are not a feature of the CYP evolution in the parasite. However, a small number of *H. contortus* genes do cluster with some of the large *C. elegans* CYP family blooms and these CYPs, particularly those expressed in the parasite gut, may merit further investigation as candidate xenobiotic metabolising genes.

The *H. contortus* genome encodes a large number of CYP genes. A number of full-length parasite CYPs share high identity with genes in *C. elegans* and life-stage, tissue- and sex-specific expression data support their classification as putative orthologues. The presence of *H. contortus* nuclear hormone receptors sharing similarity with *C. elegans daf-12*, *nhr-8* and *nhr-48* and the orthologue of *C. elegans* cytochrome P450 reductase, suggests key pathways in CYP transcription and electron transfer may also be conserved between the species. This in turn highlights the probability that *H. contortus* may utilise the full range of biotransformation pathways and xenobiotic responses known in insects and mammals. However, the parasite appears to lack the extensive gene duplications and expansions typical of CYPs with roles in environmental adaptation in other species.

CYP activity appears higher in the larval stages of *H. contortus* than in the adult, which may reflect a role in metabolising endogenous toxins and compounds essential for development or a greater exposure of free-living stages to environmental toxins. However, the relatively high expression of a small subset of CYP genes, as well as NADPH-cytochrome P450 reductase, suggests that CYP-catalysed metabolism is also important in the adult worm.

We developed a novel sequence tag approach to assay gene expression, which may be valuable for researchers working on other species with poor genome resources or when studying large and complex gene families, which may not be well-represented in draft assemblies.

## Appendix A Supplementary data

Supplementary Fig. S1 Quantitative real-time PCR (qPCR) screen of cytochrome P450 (CYP) expression in different *Haemonchus contortus* sexes and tissues. (A) CYP expression in adult male and female *H. contortus* (*Hc*). Three RNA replicates were performed. The ΔΔCT method was used to normalise gene expression relative to control gene *Hc-ama*. Positive bars indicate higher expression in females; negative bars indicate higher expression in males. (B) CYP expression in adult female intestine and soma. Three *Hc-ama* replicates served to normalise results using the ΔΔCT method. Positive bars indicate higher expression in the intestine; negative bars indicate higher expression in the soma. Numbers on both (A) and (B) *x*-axes relate to Hc-cyp-tag numbers.

Supplementary Fig. S2 Comparison of gene structure and amino acid alignment of *Caenorhabditis elegans* (Ce) *cyp31a2*, *cyp31a3* genes and *Haemonchus contortus* gene model HCOI01579500. (A) Relative to *C. elegans*, the *H. contortus* cytochrome P450 (CYP) gene is significantly larger with many intron insertions (shown as breaks between the solid boxes), but the conceptual translation (B) retains 57% identity (indicated by ∗) to the *C. elegans* proteins.

Supplementary Fig. S3 Comparison of gene structure and amino acid alignment of the *Caenorhabditis elegans* (Ce) *emb-8* and *Haemonchus contortus* (Hc) *emb-8* genes. (A) Relative to *C. elegans*, *H. contortus emb-8* is significantly larger with many intron insertions (shown as breaks between the solid boxes), but the conceptual translation (B) shows 69% identity (indicated by ∗) to *C. elegans* EMB-8. (C) Graphic representation of the protein family and domains predicted with Interpro. FAD, flavin adenine dinucleotide.

Supplementary data 4 This document contains Supplementary Tables S1 and S2.
