# Supplementary figures and images for: The cytochrome P450 family in the parasitic nematode Haemonchus contortus
Source: Int J Parasitol. 2015 Mar;45(4):243–51. doi: 10.1016/j.ijpara.2014.12.001 (PMC4365919; doi:10.1016/j.ijpara.2014.12.001)

**A**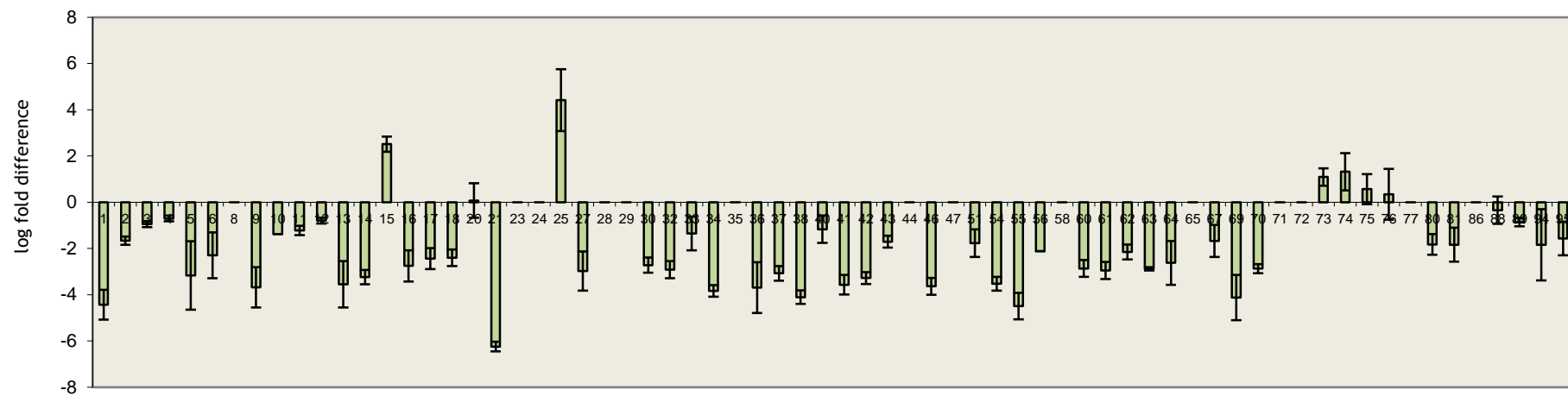**B**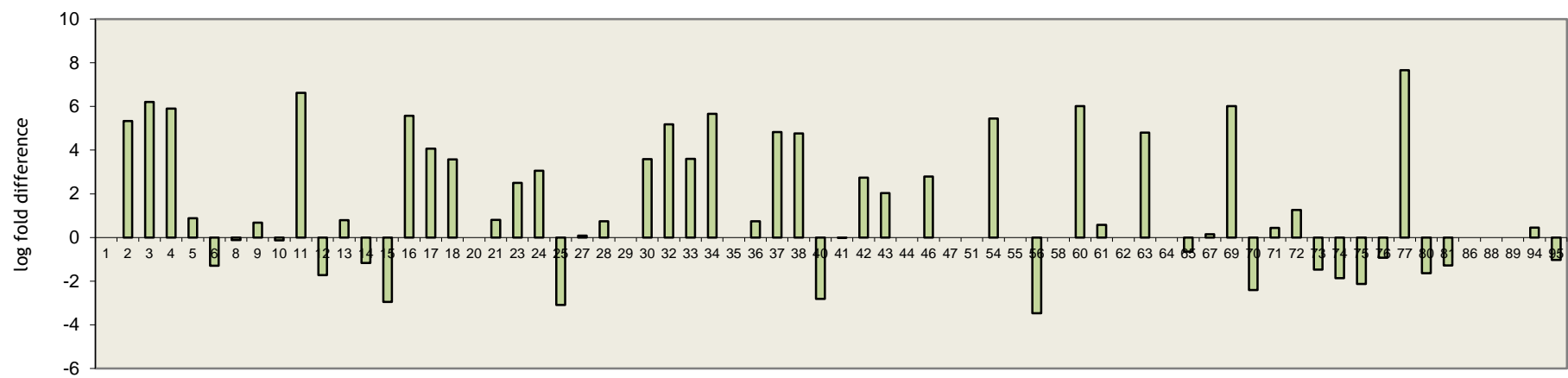

Supplement: Supplementary Fig. S1 — Quantitative real-time PCR (qPCR) screen of cytochrome P450 (CYP) expression in different Haemonchus contortus sexes and tissues. (A) CYP expression in adult male and female H. contortus (Hc). Three RNA replicates were performed. The ΔΔCT method was used to normalise gene expression relative to control gene Hc-ama. Positive bars indicate higher expression in females; negative bars indicate higher expression in males. (B) CYP expression in adult female intestine and soma. Three Hc-ama replicates served to normalise results using the ΔΔCT method. Positive bars indicate higher expression in the intestine; negative bars indicate higher expression in the soma. Numbers on both (A) and (B) x-axes relate to Hc-cyp-tag numbers. [file mmc1.pdf]
