# Supplementary material for: The cytochrome P450 family in the parasitic nematode Haemonchus contortus
Source: Int J Parasitol. 2015 Mar;45(4):243–51. doi: 10.1016/j.ijpara.2014.12.001 (PMC4365919; doi:10.1016/j.ijpara.2014.12.001)
Supplement: Supplementary Fig. S2 — Comparison of gene structure and amino acid alignment of Caenorhabditis elegans (Ce) cyp31a2, cyp31a3 genes and Haemonchus contortus gene model HCOI01579500. (A) Relative to C. elegans, the H. contortus cytochrome P450 (CYP) gene is significantly larger with many intron insertions (shown as breaks between the solid boxes), but the conceptual translation (B) retains 57% identity (indicated by ∗) to the C. elegans proteins. [file mmc2.pdf]

A.

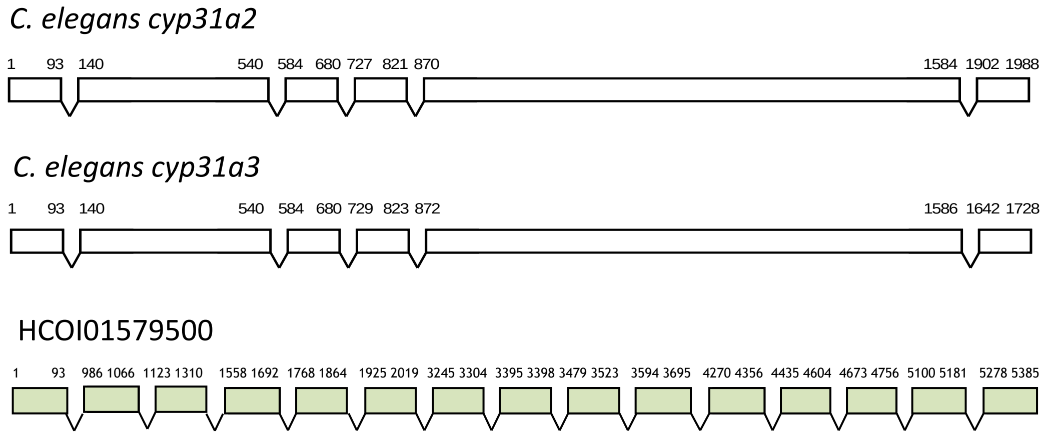

B.

```
Ce-CYP31A2      MGVIIPAVLLAMATVIAWLLYKHLRMRQVLKHLNQPRSYPIVGHGLITKPDPEGFMNQVI 60
Ce-CYP31A3      MGVIIPAVLLASATIIAWLLYKHLRMRQALKHLNQPRSYPIVGHGLVTKPDPEGFMNQVI 60
HCOI01579500    MGLLSAFLFVTVVTSIVYFIAKHLQLRNQLKGINCPRSYPLIGHGLLKKPDMEGFINQVM 60
                **:: . :::: .* *.::: ***::*: ** :* *****::****:.*** ***:***:

Ce-CYP31A2      GMGYLYPD-PRMCLLWIGPFPCLMLYSADLVEPIFSSTKHLNKGFAYVLLEPWLGISILT 119
Ce-CYP31A3      GMGYLYPD-PRMCLLWIGPFPCLMLYSADLVEPIFSSTKHLNKGFAYVLLEPWLGISILT 119
HCOI01579500    GMAQMYPDSPRMVLFWLGPVPVVMLYSARLVEKILNCSQHLNKGIAYYFFESWLGQGIIT 120
                **. :*** *** *:*:**.* :***** *** *:..::*****:** ::*.*** .*:*

Ce-CYP31A2      SQKEQWRPKRKLLTPTFHYDILKDFLPIFNEQSKILVQKLCCLGADEEVDVLSVITLCTL 179
Ce-CYP31A3      SQKEQWRPKRKLLTPTFHYDILKDFLPIFNEQSKILVQKMCSLGAEEEVDVLSVITLCTL 179
HCOI01579500    SNVDNWRPKRKLLTPTFHYDILKDFVPIFNDQAQILVKKFASLPEGKPVELMSYITLCAL 180
                *: ::********************:****:*::***:*:..*   : *:::* ****:*

Ce-CYP31A2      DIICETSMGKAIGAQLAENNEYVWAVHTINKLISKRTNNPLMWNSFIYNLTEDGRTHEKC 239
Ce-CYP31A3      DIICETSMGKAIGAQLAENNEYVWAVHTINKLISKRTNNPLMWNSFIYNLTEDGRTHEKC 239
HCOI01579500    DIICETSMGKSLNAQLDKESEYVKAVHTVNDLVQKRTKSPLYWNDYFYNKFGEGETEKKC 240
                **********::.*** ::.*** ****:*.*:.***:.** **.::**    :*.*.:**

Ce-CYP31A2      LRILHDFTKKVIVERKEALQENDYKMEGRLAFLDLLLEMVKSGQMDETDVQAEVDTFMFE 299
Ce-CYP31A3      LRILHDFTKKVIVERKEALQENDYKMEGRLAFLDLLLEMVKSGQMDETDVQAEVDTFMFE 299
HCOI01579500    IDILHSFTNKVIAERRKELEDKQWRFEGRRAFLDLLLDMANSGQLEASEIQEQVDTLMFA 300
                : ***.**:***.**:: *::::::*** *******:*.:***:: :::* :***:**

Ce-CYP31A2      GHDTTSTGLMWAIHLLGNHPEVQRKVQAELDEVMGDDEDVTIEHLSRMKYLECALKEALR 359
Ce-CYP31A3      GHDTTSTGLMWAIHLLGNHPEVQRKVQAELDEVMGDDEDVTIEHLSRMKYLECALKEALR 359
HCOI01579500    GHDTTSTGSSWALFLFGCYPEIQRKVQEEIDEVLEDSDYILPEHLPRLKYLECCLKESLR 360
                ********  **:.*:* :**:***** *:***: *.: :  ***.*:*****.***:**

Ce-CYP31A2      LFPSVPIITRELSDDQVIGGVNIPKGVTFLLNLYLVHRDPAQWKDPDVFDPDRFLPENSI 419
Ce-CYP31A3      LFPSVPIITRELSDDQVIGGVNIPKGVTFLLNLYLVHRDPSQWKDPDVFDPDRFLPENSI 419
HCOI01579500    LCTPVPMIMRKLGADQELEGVTLPKGTQVVLNQYMVHRDPMYWPDPEKFDPDRFLPENCI 420
                *  ..**:* *:*. ** : **.:***. .:** *:*****  * **: *********.*

Ce-CYP31A2      GRKSFAFIPFSAGSRNCIGQRFALMEEKVIMAHLLRNFNIKAVELMHEVRPKMEIIVRPV 479
Ce-CYP31A3      ARKSFAFIPFSAGSRNCIGQRFALMEEKVIMAHLLRNFNVKAVELMHEVRPKMEIIVRPV 479
HCOI01579500    GRHPFAFIPFSAGSRNCIGQRFGLMEIKVVVSWMLRHFNVTAVQRRCDLKSKIEIILRPQ 480
                .*:.*****************.*** **::: :**:**:.**:    :::.*:***:**

Ce-CYP31A2      TPIHMKLTRRRPIVSP------- 495
Ce-CYP31A3      TPIHMKLTRRRPIVSP------- 495
HCOI01579500    DGIHVFLEKRRAIADGFRSSLIA 503
                  **: * :**.*..
```
