# Supplementary material for: The cytochrome P450 family in the parasitic nematode Haemonchus contortus
Source: Int J Parasitol. 2015 Mar;45(4):243–51. doi: 10.1016/j.ijpara.2014.12.001 (PMC4365919; doi:10.1016/j.ijpara.2014.12.001)
Supplement: Supplementary Fig. S3 — Comparison of gene structure and amino acid alignment of the Caenorhabditis elegans (Ce) emb-8 and Haemonchus contortus (Hc) emb-8 genes. (A) Relative to C. elegans, H. contortus emb-8 is significantly larger with many intron insertions (shown as breaks between the solid boxes), but the conceptual translation (B) shows 69% identity (indicated by ∗) to C. elegans EMB-8. (C) Graphic representation of the protein family and domains predicted with Interpro. FAD, flavin adenine dinucleotide. [file mmc3.pdf]

A. *C. elegans emb-8*

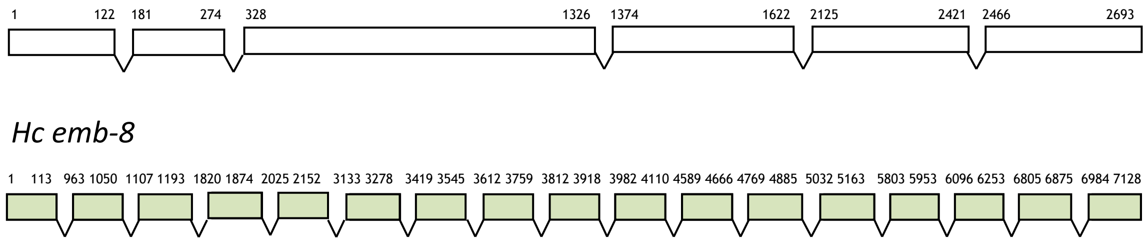

B.

```
Ce EMB-8   MLAWIVSGLDTSDLVVLTLLAGGAIIFLFMKVFNQQPSSSRYSPTVASVTTSAAASKSNQ 60
Hc EMB-8   MWDWLVSQLDVTDFIILSALMLGGMYFLVKYRLG---GTSRYKPTMTPITPTAKPK--DM 55
           *  *:** **.:*:::*: *  *.: **.   :.   .:***.**::.:*.:* ..  :

Ce EMB-8   SFIDRMKNENRQVLIMYGSQTGTAEEMSGRLAKDLTRYTKKAVVVDPEDIECEDLNRLSE 120
Hc EMB-8   SFISRMKNENRQVLIMYGSQTGTAEELSGRLAKDVSRYAKKALIVDPEEIDVDDMARLSE 115
           ***.**********************:*******::**:***::****:*: :*: ****

Ce EMB-8   VEDALLVLCIATYGEGDPTDNAVTLVEYLNAGDCDLSGVRFAVFGLGNKTYEHFNEIGIQ 180
Hc EMB-8   IKDCLVVFCMATYGEGDPTDNAQQLYEYITTTDTDFKGVNYAVFGLGNKTYEHFNAVGKL 175
           ::*.*:*:*:************  * **:.: * *:.**.:************** :*

Ce EMB-8   MDKQLEKLGAKRIFHLGLGDDDANLEEDFMIWREAFLPKVAEEFGWELNTEAETMRQYQL 240
Hc EMB-8   FDKKLEELGAERAFPLGLGDDDANLEEDFMRWREAFLPTVAQRFGWELNTDAETLRQYRL 235
           :**:**:***:* * *************** *******.**:.*******:***:***:*

Ce EMB-8   EPVEEGK--ALFKGEFGRLGAYERPRPPFDVKNPYLATVAINDELHTEHSDRSCRHIEFS 298
Hc EMB-8   ELVDHNANVTLFKGEYGRLGAFERLRPPFDQKNPFPATIAVNRELHTEKSERSCRHIEFA 295
           * *:..   :*****:*****:** ***** ***: **:*:* *****:*:********:

Ce EMB-8   VEGSRIRYEAGDHLAVFPTNDPVLVDRLINMLQFDPDHAFRLVNVDEDASKRHPFPCPTT 358
Hc EMB-8   VEGSRIRYEAGDHLAVFPTNDPELVDAVISLLDFDPEQAFRLINVDEESSKRNPFPCPCT 355
           ********************** *** :*.:*:***::****:****::***:***** *

Ce EMB-8   FRTALSHYVDICAPVKSHVLKAISEYCTDDTEKEFLNKLSTANEEGLKEYARYIVKERRS 418
Hc EMB-8   YRTALTHYVDICAPLKSHVLKAISEYCSDEKEKAYLQLLSTATEEGLKEYSSYIVKERRS 415
           :****:********:************:*:.** :*: ****.*******: ********

Ce EMB-8   IVDVLTDQKTCKPPIEYLLELLPRLQARYYSIASSPRLNEEKIAICAVVTKYSIGDRDIN 478
Hc EMB-8   IIDVLRAHPSCKPPIEYLLELLPRLQARYYSIASSPKHQENRIAICCIVTKYTIGDRLIK 475
           *:***  : :*************************: :*::****.:****:**** *:

Ce EMB-8   GVCTRYLTTKDAGSKSPVFVRKSTMRLPHRTTTQVIMIGPGTGFAPFRGFLQDRQFHKNA 538
Hc EMB-8   GVCTNYLAGKEVNDRTPVFVRKSQMRLPHRTNTPVIMIGPGTGFAPFRAFLQERKFQKDQ 535
           ****.**: *:...::******* *******.* **************.***:*:*:*:

Ce EMB-8   GKEIGAMHLYYGCRHPDHDYIYKDELAKFQEDEVLTHLVCAFSRAQEHKIYVQDRLWETR 598
Hc EMB-8   GKEIGPIMLYYGCRHPEHDYIYQDEIEEMVKDGVITDLYCAFSRAQEHKIYVQNRLWESR 595
           *****.: ********:*****:**: :: :* *:*.* ***************:****:*

Ce EMB-8   DRIWDAINVGAHVYICGDARNMARDVQATLQKIFREIGGKSETEAVAYFKDMEKTKRYQA 658
Hc EMB-8   DKVWSAIEAGAHIYVCGDARNMARDVQNVLLRILQEVGGKSEEESATLFKNLERQRRYQA 655
           *::*.**:.***:*:************ .* :*::*:***** *:.: **::*: :****

Ce EMB-8   DVWS 662
Hc EMB-8   DVWS 659
           ****
```

C. Hc EMB-8

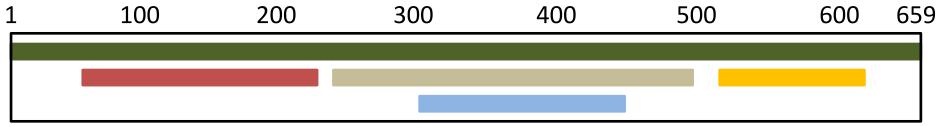

NADPH-cytochrome P450 reductase family
Flavoprotein-like domain, flavodoxin/nitric oxide synthase domain
Riboflavin synthase-like beta barrel, ferredoxin reductase-type FAD-binding domain
NADPH-cytochrome P450 reductase, FAD-binding, alpha-helical domain
Oxidoreductase FAD/NAD(P)-binding domain
