# Supplementary material for: The cytochrome P450 family in the parasitic nematode Haemonchus contortus
Source: Int J Parasitol. 2015 Mar;45(4):243–51. doi: 10.1016/j.ijpara.2014.12.001 (PMC4365919; doi:10.1016/j.ijpara.2014.12.001)
Supplement: Supplementary data 4 — This document contains Supplementary Tables S1 and S2. [file mmc4.docx]

Supplementary Table S1. Manual curation of predicted gene models for cytochrome P450s (CYPs) in *Haemonchus contortus*.

| **Locus name** | **Comment** | **Curation** |
| --- | --- | --- |
| HCOI01579500  HCOI00827700  HCOI01928800 | Truncated gene model  Two genes joined  Duplication of HCOI01929700  Two CYP genes joined | Joined HCOI01579600 to 3' end  Removed upstream gene from 5' end  Excluded HCOI01929700 from analysis  Split HCOI01928800a and HCOI01928800b |

Supplementary Table S2. Quantitative real-time PCR (qPCR) primers and typical reaction efficiencies for adult *Haemonchus contortus* cDNA. An asterisk indicates the efficiency was calculated from L3 data due to lack of adult expression.

| **Primer** | **Sequence (5’-3’)** | **Tm (^o^C)** | | **cDNA Product (bp)** | **Efficiency (%)** |
| --- | --- | --- | --- | --- | --- |
| ***Hc-ama****^a^* |  |  | |  |  |
| Hc-ama-F2 | tatgggaggtcgtgaaggtc | 60 | | 214 | 101.1 |
| Hc-ama-R2 | gtgggcttcatagtgggcata | 61 | | 214 |  |
| **Hc-cyp-tag1** |  |  | |  |  |
| Hc-cyp-tag1F1 | actcgattactggaagcggc | 60 | | 178 | 98.9 |
| Hc-cyp-tag1R1 | tcgctgattacaatcgtccgt | 60 | | 178 |  |
| **Hc-cyp-tag2** |  |  | |  |  |
| Hc-cyp-tag2F1 | cgtgtcgtcacttctagcct | 60 | | 204 | 100.9 |
| Hc-cyp-tag2R1 | gcgaatcatatcaacatcgctg | 60 | | 204 |  |
| **Hc-cyp-tag3** |  |  | |  |  |
| Hc-cyp-tag3F1 | tgagatttctccactgctgtg | 60 | | 184 | 93.5 |
| Hc-cyp-tag3R1 | cttctacgtcgtctccccat | 60 | | 184 |  |
| **Hc-cyp-tag4** |  |  | |  |  |
| Hc-cyp-tag4F2 | ggaacctgcattgtcatcga | 60 | | 181 | 99.8 |
| Hc-cyp-tag4R2 | atctccataagtgcgaatcgc | 60 | | 181 |  |
| **Hc-cyp-tag5** |  |  | |  |  |
| Hc-cyp-tag5F1 | ctgaaacgacggtgaggtct | 60 | | 135 | 98.0 |
| Hc-cyp-tag5R1 | gcaatgatgtcataccgcctga | 60 | | 135 |  |
| **Hc-cyp-tag6** |  |  | |  |  |
| Hc-cyp-tag6F1 | tggactgaatcgacggagag | 60 | | 167 | 97.6 |
| Hc-cyp-tag6R1 | agcggaatcacctcggtatc | 60 | | 167 |  |
| **Hc-cyp-tag8** |  |  | |  |  |
| Hc-cyp-tag8F1 | gtcacaaagcatttaacgcgg | 60 | | 132 | 71.2 |
| Hc-cyp-tag8R1 | tctgctgtactcgaggattct | 60 | | 132 |  |
| **Hc-cyp-tag9** |  |  | |  |  |
| Hc-cyp-tag9F1 | gagagctgaaacatccctatg | 60 | | 196 | 98.2 |
| Hc-cyp-tag9R2 | aacgcacacggagatgtcg | 60 | | 235 |  |
| **Hc-cyp-tag10** |  |  | |  |  |
| Hc-cyp-tag10F2 | cctgccgatcaacctgcta | 60 | | 167 | 31.1 |
| Hc-cyp-tag10R2 | ccgtcgtccgtaaggaatc | 60 | | 167 |  |
| **Hc-cyp-tag11** |  |  | |  |  |
| Hc-cyp-tag11F1 | tctccttctgcacattccaca | 60 | | 195 | 95.9 |
| Hc-cyp-tag11R1 | acgtgctcatccaagaccg | 60 | | 195 |  |
| **Hc-cyp-tag12** |  |  | |  |  |
| Hc-cyp-tag12F1 | aaggcgttcatgttcgttctg | 60 | | 194 | 97.3 |
| Hc-cyp-tag12R1 | tttccgatatgcactgctcca | 60 | | 194 |  |
| **Hc-cyp-tag13** |  |  | |  |  |
| Hc-cyp-tag13F1 | cttactgggtgaaacgaggc | 60 | | 126 | 101.0 |
| Hc-cyp-tag13R1 | ccgtaggtatttccatattctgc | 58 | | 126 |  |
| **Hc-cyp-tag14** |  |  | |  |  |
| Hc-cyp-tag14F1 | cgcttacttgctgtatcatcc | 60 | | 166 | 94.0 |
| Hc-cyp-tag14R1 | ggaatagcccatggtagaaca | 60 | | 166 |  |
| **Hc-cyp-tag15** |  |  | |  |  |
| Hc-cyp-tag15F2 | tgcgtaaattgggcgctgatc | 61 | | 159 | 110.0 |
| Hc-cyp-tag15R2 | gacgaccgatacagttctcag | 61 | | 159 |  |
| **Hc-cyp-tag16** |  |  | |  |  |
| Hc-cyp-tag16F3 | tgctgggatggagactacct | 60 | | 162 | 107.5 |
| Hc-cyp-tag16R3 | gcattggtgtaaggcagacg | 60 | | 162 |  |
| **Hc-cyp-tag17** |  |  | |  |  |
| Hc-cyp-tag17F3 | ggaaacgaaggaatctaccac | 60 | | 209 | 96.5 |
| Hc-cyp-tag17R3 | agcaccatccttgactaacgt | 60 | | 209 |  |
| **Hc-cyp-tag18** |  |  | |  |  |
| Hc-cyp-tag18F1 | cgtcgatacatgaggaaatgga | 60 | | 190 | 98.3 |
| Hc-cyp-tag18R1 | tgcctgacgggatatggtag | 60 | | 190 |  |
| **Hc-cyp-tag20** |  |  | |  |  |
| Hc-cyp-tag20F1 | gctacggctcactccatca | 60 | | 179 | 94.7 |
| Hc-cyp-tag20R1 | tggatagtattcttcgggacg | 60 | | 179 |  |
| **Hc-cyp-tag21** |  |  | |  |  |
| Hc-cyp-tag21F1 | ggttcgtcagatcgcagttg | 60 | | 191 | 106.5 |
| Hc-cyp-tag21R1 | cgtgaatcatggcaggcgt | 60 | | 191 |  |
| **Hc-cyp-tag23** |  |  | |  |  |
| Hc-cyp-tag23F1 | cgaccaggaccaagccc | 60 | | 201 | 83.6* |
| Hc-cyp-tag23R1 | tcgccttgttagcttcttgaaa | 58 | | 201 |  |
| **Hc-cyp-tag24** |  |  | |  |  |
| Hc-cyp-tag24F1 | cgtaatcgttggcagcgtga | 60 | | 186 | 94.2 |
| Hc-cyp-tag24R1 | cttctctccggtctttctcc | 60 | | 186 |  |
| **Hc-cyp-tag25** |  |  | |  |  |
| Hc-cyp-tag25F1 | gctgtgcatactgtcaacgat | 60 | | 170 | 104.1 |
| Hc-cyp-tag25R1 | cctgctcctggatctcgc | 61 | | 170 |  |
| **Hc-cyp-tag27** |  |  | |  |  |
| Hc-cyp-tag27F1 | gaggttcgctgtgatggaag | 60 | | 150 | 100.8 |
| Hc-cyp-tag27R1 | tcgcctttctataatcagtggg | 60 | | 150 |  |
| **Hc-cyp-tag28** |  |  | |  |  |
| Hc-cyp-tag28F1 | aatacggtcccgtccatactt | 60 | | 181 | 70.3 |
| Hc-cyp-tag28R1 | gcaaagcgacgctgttctac | 60 | | 181 |  |
| **Hc-cyp-tag29** |  |  | |  |  |
| Hc-cyp-tag29F1 | gctgtggctaccgtatccta | 60 | | 187 | 98.7* |
| Hc-cyp-tag29R1 | cagcgtatggagtgtgaatc | 58 | | 187 |  |
| **Hc-cyp-tag30** |  |  | |  |  |
| Hc-cyp-tag30F1 | ggctggtatggagacgact | 60 | | 165 | 95.5 |
| Hc-cyp-tag30R1 | tcacccctctaaccgaagtat | 60 | | 165 |  |
| **Hc-cyp-tag32** |  |  | |  |  |
| Hc-cyp-tag32F1 | cctgccctgattctgttcct | 60 | | 195 | 84.3 |
| Hc-cyp-tag32R1 | ggaagccagacagtgaagac | 60 | | 195 |  |
| **Hc-cyp-tag33** |  |  | |  |  |
| Hc-cyp-tag33F2 | tattacggatttcgatggtgtc | 58 | | 120 | 91.5* |
| Hc-cyp-tag33R2 | ccatcaagttcttccccattc | 60 | | 120 |  |
| **Hc-cyp-tag34** |  |  | |  |  |
| Hc-cyp-tag34F1 | cgatagaaatgacaaggcagac | 60 | | 163 | 102.0 |
| Hc-cyp-tag34R1 | aaggtgtcagctgggatagg | 60 | | 163 |  |
| **Hc-cyp-tag35** |  |  | |  |  |
| Hc-cyp-tag35F1 | tggtttggctgtactatgagaa | 60 | | 178 | 98.0 |
| Hc-cyp-tag35R1 | cagctataaccacatgcggc | 60 | | 178 |  |
| **Hc-cyp-tag36** |  |  | |  |  |
| Hc-cyp-tag36F1 | tactcggcgagatccacca | 60 | | 169 | 105.2* |
| Hc-cyp-tag36R1 | ccaacgcctctcctgcacat | 60 | | 169 |  |
| **Hc-cyp-tag37** |  |  | |  |  |
| Hc-cyp-tag37F1 | ccttgatttatggtttgctggg | 60 | | 176 | 91.6 |
| Hc-cyp-tag37R1 | agcattggtgtaaggcagact | 60 | | 176 |  |
| **Hc-cyp-tag38** |  |  | |  |  |
| Hc-cyp-tag38F1 | atatggcgtcgtggaaaccg | 60 | | 136 | 103.6 |
| Hc-cyp-tag38R1 | ataactgatccaacggccact | 60 | | 136 |  |
| **Hc-cyp-tag40** |  |  | |  |  |
| Hc-cyp-tag40F1 | gaatgtctgcctggacctgt | 60 | | 173 | 69.9 |
| Hc-cyp-tag40R1 | atggaggtgattcttgtcagc | 60 | | 173 |  |
| **Hc-cyp-tag41** |  |  | |  |  |
| Hc-cyp-tag41 | gcccttgccatatctgttttc | 60 | | 212 | 99.3 |
| Hc-cyp-tag41 | aaactcttgtcatcctgtctcg | 60 | | 212 |  |
| **Hc-cyp-tag42** |  |  | |  |  |
| Hc-cyp-tag42F1 | acactaagcgctatcccaaag | 60 | | 182 | 95.9 |
| Hc-cyp-tag42R1 | aatgcctctttgacaccatcg | 60 | | 182 |  |
| **Hc-cyp-tag43** |  |  | |  |  |
| Hc-cyp-tag43F1 | gccatagctaacggaactcac | 61 | | 171 | 106.4 |
| Hc-cyp-tag43R1 | gtgatagtagtttcttgaccagc | 61 | | 171 |  |
| **Hc-cyp-tag44** |  |  | |  |  |
| Hc-cyp-tag44F2 | ggtctaattgaaggaaacggg | 60 | | 184 | 81.4* |
| Hc-cyp-tag44R2 | ctagccaccacagtaaaac | 60 | | 184 |  |
| **Hc-cyp-tag46** |  |  | |  |  |
| Hc-cyp-tag46F1 | ggaccacctccttacccatt | 60 | | 165 | 103.1 |
| Hc-cyp-tag46R1 | acgaatgcctctttgactcca | 60 | | 165 |  |
| **Hc-cyp-tag47** |  |  | |  |  |
| Hc-cyp-tag47F1 | cccgttgccacggtacag | 61 | | 160 | 97.7 |
| Hc-cyp-tag47R1 | tcgtcgctgctccgtcca | 61 | | 160 |  |
| **Hc-cyp-tag51** |  |  | |  |  |
| Hc-cyp-tag51F2 | gtgggatcgtgagcggc | 60 | | 171 | 80.5 |
| Hc-cyp-tag51R2 | ggtttcctatgcgggtcc | 60 | | 171 |  |
| **Hc-cyp-tag54** |  |  | |  |  |
| Hc-cyp-tag54F1 | gtttgctgcccttattctgttc | 60 | | 199 | 101.9 |
| Hc-cyp-tag54R1 | ggaagccagacagtgaagac | 60 | | 199 |  |
| **Hc-cyp-tag55** |  |  | |  |  |
| Hc-cyp-tag55F1 | aactgttgacgcccactttc | 58 | | 169 | 114.0 |
| Hc-cyp-tag55R1 | ccgcttcgcagatgacatct | 60 | | 169 |  |
| **Hc-cyp-tag56** |  |  | |  |  |
| Hc-cyp-tag56F1 | gacctgtggatcgctggaa | 60 | | 158 | 104.5 |
| Hc-cyp-tag56R1 | ggaggtaagtcttgtcagcc | 60 | | 158 |  |
| **Hc-cyp-tag58** |  |  | |  |  |
| Hc-cyp-tag58F1 | gctacaaactgcgaaagggt | 58 | | 176 | 101.2 |
| Hc-cyp-tag58R1 | ctcccaagcacgctcgtt | 58 | | 176 |  |
| **Hc-cyp-tag60** |  |  | |  |  |
| Hc-cyp-tag60F1 | ccgtcagagaggatgtgga | 60 | | 153 | 100.1 |
| Hc-cyp-tag60R1 | catcgaagagttgtggctgta | 60 | | 153 |  |
| **Hc-cyp-tag61** |  |  | |  |  |
| Hc-cyp-tag61F2 | gtcgcgctccctttcatc | 58 | | 173 | 97.9 |
| Hc-cyp-tag61R2 | gcttcgcattcttctggtcc | 60 | | 173 |  |
| **Hc-cyp-tag62** |  |  | |  |  |
| Hc-cyp-tag62F1 | cttctacttggctggtatgga | 60 | | 158 | 96.4 |
| Hc-cyp-tag62R1 | aggtaatttctgccgatctgac | 60 | | 158 |  |
| **Hc-cyp-tag63** |  |  | |  |  |
| Hc-cyp-tag63F1 | cgtcttcactgtctggcttc | 60 | | 188 | 102.0 |
| Hc-cyp-tag63R1 | cgccgttgttccttccaga | 60 | | 188 |  |
| **Hc-cyp-tag64** |  |  | |  |  |
| Hc-cyp-tag64F1 | cttcacggcttactcgatcaa | 60 | | 182 | 97.3* |
| Hc-cyp-tag64R1 | gacttctcgcgatgctcct | 60 | | 182 |  |
| **Hc-cyp-tag65** |  |  | |  |  |
| Hc-cyp-tag65F1 | cggcgacgacttccacc | 60 | | 173 | 79.2* |
| Hc-cyp-tag65R1 | cgacggtaaatctccaaccat | 60 | | 173 |  |
| **Hc-cyp-tag67** |  |  | |  |  |
| Hc-cyp-tag67F1 | cagcaggaaaatcgtcttacag | 60 | | 166 | 106.6 |
| Hc-cyp-tag67R1 | accgattacttccttgacctct | 60 | | 166 |  |
| **Hc-cyp-tag69** |  |  | |  |  |
| Hc-cyp-tag69F1 | atcggttcagttcctgttcca | 60 | | 187 | 108.1 |
| Hc-cyp-tag69R1 | cattccttctccgacgcac | 60 | | 187 |  |
| **Hc-cyp-tag70** |  |  | |  |  |
| Hc-cyp-tag70F1 | ggatatgaacgcaaaaggagag | 60 | | 166 | 112.3 |
| Hc-cyp-tag70R1 | ctcatcgacttcacgttgtac | 60 | | 166 |  |
| **Hc-cyp-tag71** |  |  | |  |  |
| Hc-cyp-tag71F1 | cactttacggacattacggga | 60 | | 188 | 70.5 |
| Hc-cyp-tag71R1 | gattcgattgataacactgccg | 60 | | 188 |  |
| **Hc-cyp-tag72** |  |  | |  |  |
| Hc-cyp-tag72F1 | tcgaaccaaggaaggcaaac | 60 | | 201 | 110.2 |
| Hc-cyp-tag72R1 | caccgagacatgaccgtttc | 60 | | 201 |  |
| **Hc-cyp-tag73** |  |  | |  |  |
| Hc-cyp-tag73F1 | aggtggctcaggaattggtg | 60 | | 161 | 109.5 |
| Hc-cyp-tag73R1 | agcccattcaatgcgatcagt | 60 | | 161 |  |
| **Hc-cyp-tag74** |  |  | |  |  |
| Hc-cyp-tag74F1 | cccctatgcttgtctacaac | 58 | | 201 | 93.9 |
| Hc-cyp-tag74R1 | atatctactggtgtgccagc | 58 | | 201 |  |
| **Hc-cyp-tag75** |  |  | |  |  |
| Hc-cyp-tag75F1 | gcttctcgccagcaggattt | 60 | | 176 | 110.3 |
| Hc-cyp-tag75R1 | aagtgtctcataggtctgttcg | 60 | | 176 |  |
| **Hc-cyp-tag76** |  |  | |  |  |
| Hc-cyp-tag76F1 | ctcgcattccaaccaagtagat | 60 | | 206 | 83.3* |
| Hc-cyp-tag76R1 | gtgtctcataggtctgttcgat | 60 | | 206 |  |
| **Hc-cyp-tag77** |  |  | |  |  |
| Hc-cyp-tag77F1 | tctctccttctgcacattctac | 60 | | 195 | 94.5 |
| Hc-cyp-tag77R1 | tgctcatccgaaaccggg | 58 | | 195 |  |
| **Hc-cyp-tag80** |  |  | |  |  |
| Hc-cyp-tag80F1 | ggccagaggtttgctgtgat | 60 | | 162 | 101.0 |
| Hc-cyp-tag80R1 | ctcagttcgcttttcaataatcag | 60 | | 162 |  |
| **Hc-cyp-tag81** |  |  | |  |  |
| Hc-cyp-tag81F1 | gcgacagattacgtggcag | 60 | | 154 | 107.5 |
| Hc-cyp-tag81R1 | ctccccatgaaagcgtgttg | 60 | | 154 |  |
| **Hc-cyp-tag86** |  |  | |  |  |
| Hc-cyp-tag86F1 | tgagtggatacggtttcgagt | 60 | | 186 | 105.7 |
| Hc-cyp-tag86R1 | cctcatcactcaactggttag | 60 | | 186 |  |
| **Hc-cyp-tag88** |  |  | |  |  |
| Hc-cyp-tag88F1 | caagaagtctatcgtgctggc | 58 | | 167 | 107.6 |
| Hc-cyp-tag88R1 | atgattagttccggtcgagg | 58 | | 167 |  |
| **Hc-cyp-tag89** |  |  | |  |  |
| Hc-cyp-tag89F1 | ggaggaatggggacgacaat | 60 | | 147 | 102.8 |
| Hc-cyp-tag89R1 | ccggttcctatccaatctga | 58 | | 147 |  |
| **Hc-cyp-tag94** |  |  | |  |  |
| Hc-cyp-tag94F1 | gaactggagagaacaacgtc | 58 | | 184 | 103.2 |
| Hc-cyp-tag94R1 | ctcgtttgcagcgtccatca | 60 | | 184 |  |
| **Hc-cyp-tag95** |  |  | |  |  |
| Hc-cyp-tag95F1 | gaatctactccacgaaacactt | 58 | | 202 | 104.2 |
| Hc-cyp-tag95R1 | gcccaagcactgacgtttc | 60 | | 202 |  |
|  |  |  | |  |  |
| *^a^ Haemonchus contortus* homologue of *Caenorhabditis elegans ama-1*, which encodes a subunit of RNA polymerase II. | | | | | |
|  |  | |  |  |  |
| Tm, melting temperature; Hc-cyp-tag, *H. contortus* cytochrome P450 tag. | | | | | |
